# Hospitals in India

**Published:** 1893-10-28

**Authors:** 


### HOSPITALS IN INDIA.

### I.—THE BENGAL PRESIDENCY.

#### (3).—Dispensaries in the Punjab.

In 1891 there were in the Junjab 228 hospitals and dispensaries, classified as follows:—

| | |
|---|---|
| State Hospital | 1 |
| State Dispensaries, Class I. | 3 |
| Local Fund Institutions, Class II. | 218 |
| Private Institutions, Class III. A | 2 |
| State-aided Private Institutions, Class III. B ... | 4 |
| Total | 228 |

In addition to these there are 19 canal dispensaries, from which no returns are received. There is one dispensary in the Province to every 90,000 of the approximate population, which compares favourably with the North-Western Provinces, where there is one to every 160,000 of population, and with Lower Bengal, where there is one to every 270,000. The number of beds entered in the returns as available was 3,005, of which 926 were for women; but we are warned that the numbers are to a great extent fictitious, the accommodation being insufficient and the dispensaries in rural districts being not properly equipped for the treatment of indoor patients, although the actual beds no doubt exist.

The daily average number of beds occupied was 1,660—by men, 1,264; by women, 283; and by children, 113. The total number of indoor patients was 49,700, of whom 33,682 were cured, 7,185 were relieved, 4,812 were discharged "otherwise," and 2,678 died. The out-patients numbered 2,541,753, with an average daily attendance of 14,971·16. Indoor and outdoor patients combined thus numbered 2,591,453, giving a percentage of 12·5 on the approximate population, as compared with about 6 and 2 per cent. in the North-Western Provinces and Lower Bengal respectively. With regard to the diseases for which relief was sought, the returns show that 3,988 cases of cholera were treated, or nearly 1,300 more than in 1890, whereas the cases of malarial fever numbered only 421,677, against 580,273 in the previous year. This goes to show that the year was a healthier one, but, on the other hand, the malarial influences prevailing in 1890 left their mark on 1891 by an increase of cases of scurvy, debility, digestive system diseases, spleen, and ulcer. There were 185,120 surgical operations performed during the year, of which 13,736 were major. Taking into account 318 cases remaining from the previous year, the surgical returns show that of the total of 14,054 major operations performed 11,339 were cured, 1,438 were relieved, 646 were discharged "otherwise," and 293 died, leaving 338 under treatment at the close of the year.

Referring to the financial statements appended to the report under consideration, we quote the following summary of the receipts of the dispensaries for 1891, giving those for 1890 as well for purposes of comparison:—

Table Showing the Dispensary Receipts for 1890 and 1891.

| | 1891. Rs. | a. | p. | 1890. Rs. | a. | p. |
|---|---|---|---|---|---|---|
| From Municipal Committees... | 216,361 | 11 | 0 | 190,051 | 7 | 10 |
| From District Boards and Local Sources ... | 208,434 | 2 | 0 | 205,406 | 8 | 7 |
| From Provincial Funds ... | 17,219 | 0 | 7 | 32,719 | 15 | 3 |
| Subscriptions from Europeans | 15,835 | 2 | 8 | 19,906 | 5 | 0 |
| Subscriptions from Natives ... | 8,283 | 2 | 8 | 7,683 | 5 | 11 |
| Sale of European Medicines... | 9,815 | 9 | 1 | 11,084 | 1 | 4 |
| Interest on Investments ... | 5,286 | 14 | 4 | 5,393 | 11 | 10 |
| Sale of Securities ... ... | Nil. | | | 500 | 0 | 0 |
| Total ... | 481,235 | 10 | 4 | 472,745 | 7 | 9 |

The expenditure amounted to Rs.476,406 13a. 10p., made up thus: Establishments, Rs.253,210 1a. 6p.; diet, Rs.28,552 15a. 11p.; European medicines, Rs.87,339 12a. 7p.; Bazar medicines, Rs.15,137 10a. 3p.; miscellaneous, Rs.44,067 11a. 10p.; and buildings and repairs, Rs.48,098 9a. 9p. In 1890 it was Rs.465,568 7a 2p., but as the expenditure of the Mayo Hospital is, under the orders of Government, excluded from the statement for 1891, and treated separately, it may also for purposes of comparison be deducted from the figures of 1890, which reduces the expenditure of that year to Rs.438,653 15a. 9p., showing an increase in 1891 of Rs.37,752 14a. 1p. This increase is due in "establishments" to the opening of new dispensaries, and to the pay of hospital assistants on local cholera duty; in "diets" it is due to the larger number of indoor patients treated.

In conclusion, we must make a brief reference to the leper asylums in the Punjab. There are six of these institutions, with a total population of 605 lepers in 1891. Of this number 123 left of their own accord and 71 died, leaving 411 in residence at the end of the year. The mortality on total population was at the rate of 117 per thousand. It seems strange that the Leprosy Commission should not insist upon the sexes being kept apart, and we strongly recommend they should be separated. Since the foundation of one asylum 30 unfortunate children have been born of leper parents. Of these thirteen died in childhood, one being a leper; five left the asylum, one a leper; and of the twelve now in the institution six are lepers. The total income of these asylums was Rs.21,385, and the gross expenditure was Rs.21,165, leaving a credit balance of Rs. 220.

